# Osteoprotegerin (OPG) as a biomarker for diabetic cardiovascular complications

**DOI:** 10.1186/2193-1801-2-658

**Published:** 2013-12-06

**Authors:** Mette Bjerre

**Affiliations:** The Medical Research Laboratory, Department of Clinical Medicine, Faculty of Health, Aarhus University, Nørrebrogade 44, Building 3b, DK-8000 Aarhus C, Denmark

**Keywords:** Osteoprotegerin, OPG, Diabetes, Cardiovascular disease, Biomarker

## Abstract

Osteoprotegerin (OPG) is a glycoprotein involved in bone metabolisms and with a regulatory role in immune, skeletal and vascular systems. Recently, circulating OPG levels have emerged as independent biomarkers of cardiovascular disease (CVD) in patients with acute or chronic heart disease, as well as in the healthy population. Furthermore, OPG has been implicated in various inflammations and linked to diabetes and poor glycaemic control. This review focuses on the relations between circulating OPG levels and cardiovascular complications, with special emphasis on diabetic patients. OPG levels were observed to increase concurrently with the severity of diabetic complications, that is, with the highest circulating OPG levels observed in diabetic patients dying from CVD. Although the clinical prognostic use of OPG may seem far away, OPG does look promising as a biomarker in order to help the cardiologist to a better risk-stratification of the patients.

## Osteoprotegerin (OPG)

In 1997 (Simonet et al. 1997) characterized osteoprotegerin (OPG) (also known as Osteoclastogenesis Inhibitory Factor (OCIF) (Yamaguchi et al. 1998) or tumour necrosis factor receptor superfamily member 11b (TNFRSF11B)) as a secreted glycoprotein that regulates bone resorption. OPG is synthesized as a monomer (60 kDa) and assembled as a homodimer within the cell, and then secreted mainly as a disulphide-linked homodimer into the circulation (Yamaguchi et al. 1998; Simonet et al. 1997). OPG was identified as a cytokine and member of the TNF receptor superfamily, and binds to two ligands, RANKL (receptor activator of nuclear factor kB ligand), a critical cytokine for osteoclast differentiation, and TRAIL (TNF-related apoptosis-inducing ligand), involved in immune surveillance (Emery et al. 1998; Schoppet et al. 2002). Thus, acting as a decoy receptor for RANKL and TRAIL, OPG inhibits the nuclear factor-kB’s regulatory effects on inflammation, skeletal, and vascular systems and prevents TRAIL-induced apoptosis.

The OPG molecule consists of three structural domains influencing the biological function. The N-terminal part is a cysteine-rich domain important for dimerization and osteoclastgenesis whereas the C-terminal contains a death domain and a domain for heparin binding (Yamaguchi et al. 1998). The latter is capable of interacting with different proteoglycans including heparan sulphate and heparin (Theoleyre et al. 2006). Binding between OPG and heparan sulphate present at the cell surface has been reported in myeloma cells (Standal et al. 2002) and human monocytes (Mosheimer et al. 2005). OPG is highly expressed in heart, lung, kidney, liver and bone marrow among other tissues (Simonet et al. 1997) and produced by vascular endothelial and smooth muscle cells (SMC) and secreted into the circulation (Hofbauer et al. 2001). OPG is found in the Weibel Palade bodies and in platelets where it is associated with von Willebrandt factor (Zannettino et al. 2005; Chollet et al. 2010). TNF-α and IL-1β were found to increase the OPG levels, indicating that activation of endothelial cells by pro-inflammatory cytokines might be a possible source of circulating OPG in patients with cardiovascular disease (CVD) (Hofbauer and Schoppet 2004; Schoppet et al. 2002). *In vitro* experiments show that human SMCs produce large OPG amounts (up to 30 times more than endothelial cells) after stimulation with TNF-α, whereas insulin was found to decrease the production (Olesen et al. 2005). The effects were related to changes in mRNA indicating a transcriptional regulation.

## OPG and CVD

Lately, a connection between bone regulatory proteins and vascular biology has attracted attention, suggesting OPG as a possible mediator of vascular calcification (Flyvbjerg 2010; Reid and Holen 2009). Arterial calcification is part of the atherosclerotic process leading to clinical CVD. OPG is reported to be present in atherosclerotic plaques and studies have shown that OPG co-localise with the area of calcification (Dhore et al. 2001; Schoppet et al. 2004). Several prognostic associations involving circulating OPG levels have been reported, both regarding risk of CVD and of the subsequent risk of death (Montagnana et al. 2013). The link between OPG and CVD was further supported by observations of OPG promoter polymorphism that is related to vascular morphology and function (Brandstrom et al. 2002; Soufi et al. 2004). The clinical relevance of the polymorphisms is based on the fact that plasma OPG levels and functional activity may be influenced. Recently, three polymorphisms (T245G, T950C and G1181C) in the OPG gene, which are reported to be associated with increased serum OPG levels, were found more frequently in patients with carotid plaques (Straface et al. 2011) or in diabetic patients with a history of ischemic stroke (Biscetti et al. 2013).

Using animal models, Bucay et al. showed that OPG knockout mice developed spontaneous arterial calcification, thus OPG appears to be protective against vascular calcification (Bucay et al. 1998). Furthermore, in ApoE knockout mice, a well-known model for atherosclerosis, depletion of OPG increased atherosclerotic lesion progression and calcification (Bennett et al. 2006). Therefore, the elevated OPG levels may prevent cardiovascular events in humans. However, the protective role of OPG found in animal models has not been observed in humans. In fact, increased OPG levels have consistently been associated with the severity of CVD.

**Figure 1:**
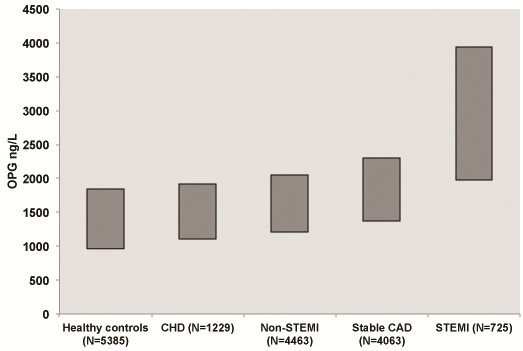
**Increasing OPG levels associate with the severity of the cardiovascular disease.** All analyses are performed in The Medical Research Laboratory (OPG levels median (IQR)) ((Mogelvang et al. 2012; Roysland et al. 2010; Roysland et al. 2012; Pedersen et al. 2012) and Bjerre et al. unpublished data).

The first connection between OPG and CVD in humans was published in 2001by Browner et al. reporting an association between high OPG levels in plasma and increased CV mortality in a cohort of 490 women above the age of 65 (Browner et al. 2001). Several associations between OPG levels and traditional CV risk factors have now been reported, including positive correlations with smoking, fasting glucose levels, older age, diabetes, and renal impairment (Kiechl et al. 2006). In addition, OPG concentrations are positively correlated with coronary calcification, vascular stiffness and the presence of unstable atherosclerotic plaques (Nybo and Rasmussen 2008a; Montecucco et al. 2007). We have recently shown that increased serum OPG levels on admission for acute myocardial infarct (AMI) are associated with decreased microcirculation after revascularization (Logstrup et al. 2013). In population-based cohort studies, increased OPG levels were found to be associated with the future risk of myocardial infarct, ischemic stroke, and CV mortality (Kiechl et al. 2004; Vik et al. 2011; Mogelvang et al. 2013; Abedin et al. 2007). In an investigation of a large number of patients with different types of CVD, we found associations between increasing OPG levels and the severity of CVD (Figure 1).

Increased OPG levels have recently been associated with a greater extent of myocardial damage and lower myocardial salvage, estimated by magnetic resonance imaging or by single photon emission computed tomography (SPECT), in STEMI patients treated with pPCI (Fuernau et al. 2012; Andersen et al. 2011). However, we could not confirm this in our very similar set-up (patients (N = 219), pPCI and SPECT imaging). Instead, our results suggest that increased circulating OPG levels are not directly involved in the myocardial damage after STEMI (*Bjerre et al. manuscript submitted*). Interestingly, we showed that high OPG levels still predicted a significantly increased risk of major CV events. The fact that OPG is present in platelets may be the link between increased OPG levels and thrombosis. Thus, the OPG levels may simply reflect extensive CVD.

## OPG, Diabetes and CVD

The initial lesion of atherosclerosis involves changes in the vascular endothelium and patients with diabetes show endothelial dysfunction as well as associated CV risk factors such as hypertension, obesity and dyslipidaemia (Versari et al. 2009). Thus, diabetes plays a critical role in the development of CVD. In fact, the mortality from AMI is increased five-fold in diabetic patients (Hansen et al. 2007). Interestingly, Redgrave et al. reported no fundamental difference between carotid plaques from diabetics and non-diabetics, but surface thrombus seems to persist longer after ischemic symptoms in plaques from diabetics or patients with impaired glucose tolerance (Redgrave et al. 2008). Hyperglycaemia accelerates atherosclerosis and increases the risk for AMI, thus worsening the prognosis in diabetics (Mazzone et al. 2008; Schramm et al. 2008). Changes in the vascular endothelium in diabetics may account for the association with multiple vascular complications. The UKPDS study reported a positive effect on the vascular system by lowering the blood glucose (Holman et al. 2008). Noteworthy, a positive correlation between fructosamine and serum OPG was found in a group of elderly women with diabetes, but not in the non-diabetic controls (Browner et al. 2001). In agreement with this, a significant correlation between HbA_1c_ and OPG was shown in patients with type 1 diabetes (T1D) or type 2 diabetes (T2D) (Knudsen et al. 2003; Rasmussen et al. 2006). Also, children with T1D had higher OPG levels than healthy age, sex and BMI matched children (Galluzzi et al. 2005), and OPG was significantly correlated with HbA_1c_. These findings were later confirmed in rodent models of experimental diabetic arteriopathy (Heinonen et al. 2007; Vaccarezza et al. 2007).

Browner et al. reported 30% higher serum OPG levels in women with diabetes as compared to healthy individuals (Browner et al. 2001), and OPG has been found to accumulate in aortic tissue from patients with T1D and T2D (Olesen et al. 2005). We have shown that serum OPG is associated with the development and progression of diabetic complications in a large group of adults with T1D (N = 1939), and that OPG is an independent predictor of CV complications (Gordin et al. 2013). In addition, patients with renal impairment had elevated OPG levels compared to T1D patients without overt kidney disease. These results correlated with the findings by Jorsal et al., showing that increased OPG levels predicted the severity of diabetic nephropathy, and that OPG is an independent marker of mortality (Jorsal et al. 2008).

Similarly, Knudsen et al. reported increased OPG levels in T2D patients with microvascular complications compared to T2D patients without complications (Knudsen et al. 2003). Anard and co-workers reported that elevated OPG levels predicted CV events in patients with uncomplicated T2D (Anand et al. 2006) and we showed increased OPG levels in patients with uncomplicated well-controlled T2D (Chen et al. 2011). In a prospective observational study with a 17-year-follow-up period, increased OPG levels were reported as a strong predictor of all-cause mortality in T2D patients, independent of traditional cardiovascular risk-factors (Reinhard et al. 2010).

**Figure 2:**
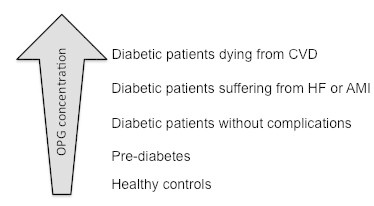
**OPG levels increase with the severity of complications in diabetic patients.** All OPG analyses are performed in The Medical Research Laboratory (Mogelvang et al. 2012; Chen et al. 2011; Gordin et al. 2013) and Bjerre et al. unpublished data).

A few studies contradicted the numerous studies described above. Identical OPG levels was observed in obese and lean individuals and in T2D patients (N = 10 in each group). However, acute hyperinsulinemia decreased OPG with diminished effect in obese individuals and T2D patients (Jorgensen et al. 2009). In addition, significantly low OPG levels were reported in T1D patients with normoalbuminuria compared with healthy controls (Singh et al. 2010). In a group of diabetic patients with stable coronary artery disease (CAD) we found significantly increased serum OPG levels as compared to the non-diabetic CAD patients (*Bjerre et al. submitted for publication*). Of notice, significantly increased OPG levels were found in the diabetic patients who died compared with surviving diabetic patients. Collectively, increasing OPG concentrations in diabetic patients seem to follow the severity of complications (Figure 2).

## OPG as a Biomarker

Taken together, these studies suggest that OPG may be a new promising marker for risk prediction in CVD. However, before implementation of OPG as a biomarker some considerations must be taken into account.

OPG can be detected in both serum and plasma, but the levels are not completely comparable, and EDTA plasma seems to be recommended (Chan et al. 2003). The OPG molecule primarily circulates as a homodimer, but also monomer or complexes with RANKL or TRAIL may be present, which may interfere with the measurements. Furthermore, the circulating OPG reflects the production from several tissues, which makes it difficult to specify the site of origin.

Of notice, the OPG molecule contains a heparin binding domain and *in vitro* studies have shown a rapid OPG release from smooth muscle cells (SMC) after heparin treatment (Nybo and Rasmussen 2008b). According to guidelines, all patients with CV complications are treated with unfractionated heparin within the ambulance. *In vivo* studies with intravenous heparin infusions in healthy individuals showed a 2.2 fold increase in the circulating OPG levels within 5 min as compared to pre-injection values, but the OPG levels were significantly decreased or normalized within 1 hour (Vik et al. 2007; Nybo and Rasmussen 2008b). The time for collection of blood samples is therefore of great importance and can make it difficult to compare studies of OPG levels in patients with acute CV events.

In addition, OPG levels are gender-specific, i.e. women have higher OPG levels than men. Furthermore, OPG is strongly associated with age (Mogelvang et al. 2013; Omland et al. 2007), which also needs to be considered before appointing threshold or risk stratification.

## Conclusion

At first glance, it may seem contradictory that OPG – a putative beneficial calcification inhibitor – accumulates in the arterial system in diabetes and that high levels of OPG are risk markers for cardiovascular death. However, the accumulation may be a consequence of a compensatory vascular response towards calcification and thus associated with endothelial dysfunction. Since the inflammatory response is a natural sequence of a plaque rupture, OPG may be up regulated to prevent further damage, rather than being responsible for the initial damage.

Despite the growing evidence, the precise mechanism by which OPG, diabetes and cardiovascular disease are connected has not yet been found and the actual role of OPG in atherosclerotic calcification remains speculative. Although the prognostic use of OPG in the clinic seems far away, it does look promising in order to help the cardiologist to a better risk-stratification of the patients.
